# Profile of graduates from the UFMG Nursing Graduate Program (2017–2020): a Freidsonian analysis

**DOI:** 10.1590/1980-220X-REEUSP-2026-0234en

**Published:** 2026-07-24

**Authors:** Bárbara Costa Moreira, Pacita Geovana Gama de Sousa Aperibense, Maria Itayra Padilha, Camila Pureza Guimarães da Silva, Maria Angélica de Almeida Peres, Fernanda Batista Oliveira Santos

**Affiliations:** 1 Universidade Federal de Minas Gerais, Escola de Enfermagem, Programa de Pós-Graduação em Enfermagem, Belo Horizonte, MG, Brazil.; 2 Universidade Federal do Rio de Janeiro, Departamento de Enfermagem Fundamental, Macaé, RJ, Brazil.; 3 Universidade Federal de Santa Catarina, Departamento de Enfermagem e do Programa de Pós-graduação em Enfermagem, Florianópolis, SC, Brazil.; 4 Universidade Federal do Rio de Janeiro, Escola de Enfermagem Anna Nery, Departamento de Enfermagem Fundamental, RJ, Brazil.

**Keywords:** Education, Graduate, Health Postgraduate Programs, Education, Nursing, Institutional Analysis, Educational Measurement

## Abstract

**Objective:**

To characterize the demographic, academic, and professional profile of graduates from the stricto sensu Graduate Program in Nursing at the School of Nursing of the Federal University of Minas Gerais during the 2017–2020 quadrennium, in light of Eliot Freidson’s sociology of professions.

**Method:**

A qualitative socio-historical study conducted according to COREQ guidelines. Data were collected between October 2024 and April 2025 from the Program’s graduate database and the Lattes Platform, analyzed through internal and external criticism based on pertinence, representativeness, and exhaustiveness, and interpreted using Eliot Freidson’s Sociology of Professions.

**Results:**

The Freidsonian analysis revealed credentialism, strengthening of professional status and autonomy, and greater insertion of PhD graduates into prestigious positions, while gender inequalities in leadership and salary disparities persisted.

**Conclusion:**

The study reaffirms the relevance of the Minas Gerais Nursing Graduate Program as a training space for critical professionals committed to science, teaching, and society. The graduates’ profile demonstrated individual and institutional impacts, consolidating the program as a national reference in academic excellence.

## INTRODUCTION

The intense industrial, economic, and political transformations that took place in Brazil during the 1960s led to the enactment of the 1968 University Reform, resulting in changes to undergraduate programs and the creation of Graduate Programs^(1,2,3)^. In Nursing, the consolidation of stricto sensu graduate education followed a non-linear trajectory, marked by the strengthening of the profession, the expansion of scientific production, the national and international consolidation of programs, and their contribution to the internationalization of Brazilian science^(4)^.

Graduate Programs are composed of faculty, technical-administrative staff, and students, whose dynamics involve multiple perspectives and experiences. The vision and mission of these programs require their social actors to engage in reflective care and management practices aimed at advancing science and technology in the Health field. Therefore, considering the development of these individuals becomes essential for understanding the social, political, and cultural impacts generated, supporting the improvement and strengthening of the programs^(4)^.

In Minas Gerais, the School of Nursing at the *Universidade Federal de Minas Gerais* (EEUFMG, Federal University of Minas Gerais) has played a central role in the professionalization of nursing since its founding in 1933. It was responsible for creating the first Stricto Sensu Graduate Program in Nursing (PPGE) in the state, established in 1993 with its first master’s cohort. The doctoral program was launched in 2004, further expanding its contribution to the training of researchers and to the scientific advancement of Brazilian nursing^(3)^.

The strengthening of graduate education in Brazil became evident beginning in 1981, with the creation of the National Stricto Sensu Graduate Plan by the Coordination for the Improvement of Higher Education Personnel (CAPES). Currently responsible for the quadrennial evaluation of graduate programs, the institution recommends that, regarding training, the trajectory of graduates be considered, including an analysis of their professional placement and the social, political, and economic impacts resulting from their qualification^(5,6)^.

Since 2020, the PPGE/UFMG has monitored its graduates after their thesis or dissertation defense and for the five subsequent years following program completion, as recommended by CAPES. Graduates are directed to the program’s website to complete a form containing information on identification data, degrees obtained, educational background, and professional activities during the data collection period.

From the perspective of the sociology of professions, it is possible to understand the consolidation of graduate education in nursing as part of a broader process of professionalization. According to Freidson, a profession is structured upon three fundamental pillars: expertise, autonomy, and credentialism^(7)^. In this sense, Graduate Programs in Nursing become important spaces for the development of a technical-scientific body of knowledge, contributing to the consolidation of nursing as an autonomous and recognized professional field. Identifying the professional trajectories of graduates is therefore essential for assessing how this training influences their insertion into the labor market, their prestige, and their technical authority, reflecting the mechanisms of reproduction and valorization of the profession in the contemporary Brazilian context.

Despite the advances of stricto sensu graduate education in Nursing in Brazil, studies that critically analyze the impacts of this training on the professional trajectories of graduates remain scarce^(8)^. The importance of investigations in this direction is reinforced by the Ministry of Health’s Research Priorities Agenda (APPMS), which highlights the need to assess the impact of educational offerings on the qualification of professionals within the *Sistema Único de Saúde* (SUS, Brazilian Unified Health System)^(9)^. Complementarily, the document “Research Priorities for Nursing: Preliminary Proposal” from the Brazilian Nursing Association (ABEn) underscores the relevance of studies on stricto sensu training, its capacity to develop competencies, improve health work, and contribute to SUS management^(10)^.

In this context, understanding the professional trajectories of PPGE/UFMG graduates from the 2017–2020 quadrennium becomes essential to support discussions on the valorization of Nursing and the strengthening of the public health system. In light of this, the following question arises: how has the professional trajectory of PPGE/UFMG graduates from 2017 to 2020 unfolded after completing their master’s and/or doctoral degrees?

This study aims to characterize the demographic, academic, and professional profile of graduates from the stricto sensu Graduate Program in Nursing at the School of Nursing of the Federal University of Minas Gerais during the 2017–2020 quadrennium, in light of Eliot Freidson’s sociology of professions.

The premise is that a stricto sensu degree in nursing represents more than an academic qualification. It functions as a mechanism of differentiation within the professional field, conferring technical authority and status upon graduates. From the perspective of the sociology of professions, it is assumed that graduate education contributes to the construction of specialized knowledge, legitimizes prestigious positions in the labor market, and reinforces processes of professionalization in nursing.

## METHOD

### Type of Study

This study is situated within the field of History, with a socio-historical dimension aimed at examining the specificities of a society, including the modes and mechanisms of social organization, the relationships among groups, institutions, and individuals, as well as the processes of social transformation^(11,12)^. The research aligns with the domains of nursing history. The approach adopted is qualitative, using Textual History and documentary analysis of written sources.

The study was conducted following the recommendations of the Consolidated Criteria for Reporting Qualitative Research (COREQ) for qualitative studies.

### Location

The study was conducted at the School of Nursing of the Federal University of Minas Gerais (EEUFMG), in Belo Horizonte, which hosts the Graduate Program in Nursing (PPGE). The temporal and spatial scope encompasses the 2017–2020 quadrennium, a period marked by a context of economic liberalism, attacks on democracy, and challenges to mechanisms of social participation. Added to this scenario was the COVID19 pandemic, which required restructuring within the health and education sectors.

### Sources and Data Collection

The textual sources that composed this study were obtained from the Graduate Program Committee (CPG) of the School of Nursing at UFMG, including the PPGE/UFMG graduate database. Searches on the Lattes Platform, maintained by the National Council for Scientific and Technological Development (CNPq), were conducted to gather data on the professional trajectories of graduates. The choice of this platform is justified by its status as Brazil’s main virtual system for scientific curricula and its open-access nature, which allows for verification of graduates’ identities.

Textual sources were included only when they were up to date and linked to the graduate’s full name. Incomplete, outdated records or those without verifiable correspondence to the program’s database were excluded.

Data were collected by the principal researcher between October 2024 and April 2025.

### Analysis and Data Processing

The treatment of the sources included the creation and organization of a database on the Google Drive platform, described and arranged by year (2017 to 2020). Once catalogued, the sources were categorized based on established methodological criteria such as pertinence, sufficiency, representativeness, exhaustiveness, and homogeneity. The textual sources were critically analyzed regarding their accuracy and relevance through internal and external criticism^(12)^. The findings were analyzed in light of Eliot Freidson’s Sociology of Professions.

### Ethical Aspects

The study was reviewed by the Research Ethics Committee of UFMG, ensuring assent and compliance with current ethical and legal regulations. It was approved under opinion no. 7,115,336 in the year 2024.

## RESULTS

In the 2017–2020 quadrennium, the Graduate Program in Nursing at the Federal University of Minas Gerais (PPGE/UFMG) awarded degrees to 218 students, including 130 master’s graduates and 88 doctoral graduates. Of the total number of graduates, 168 responded to the questionnaire, representing 77.06% of the cohort; among them, 87 were master’s graduates and 81 were doctoral graduates.

**Figure 1:**
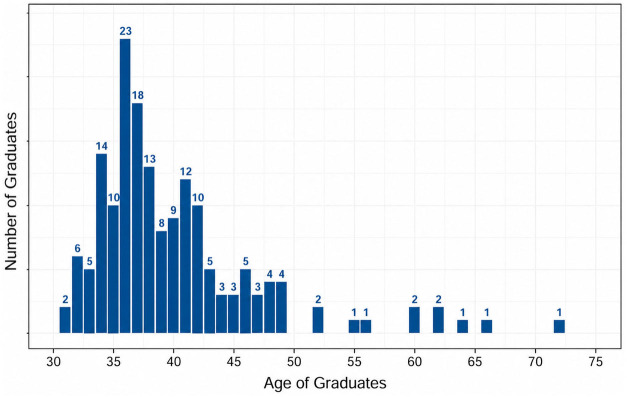
Age distribution of PPGE graduates.

Source: Statistical analysis report of graduate program alumni (2017–2020), 2025.

Of all graduates from this period, 190 were women and 28 were men, demonstrating a marked female predominance (87%). The age distribution of PPGE/UFMG graduates is shown in the graph below (Figure 1), indicating a higher concentration at age 36, with 23 graduates, followed by ages 37 (n = 18), 38 (n = 13), and 34 (n = 14).

The initial training of PPGE/UFMG graduates is predominantly composed of nurses, totaling 147 professionals. Throughout the 2017–2020 quadrennium, the program also included one Physical Education professional, one Statistician, two pharmacists, three health managers, eight nutritionists, one educator, four psychologists, and one occupational therapist.

After identifying the predominance of nurses among the graduates, a strong female presence is observed within this group. PPGE/UFMG data indicate that, between 2017 and 2020, 128 women completed the program, compared with only 19 men.

Regarding the graduates’ institutions of undergraduate education, 62 students completed their degrees at UFMG, 63 at other public universities in Brazil, and 36 at private higher education institutions.

Of the 168 graduates who responded to the CPG questionnaire, 126 (75%) had completed a lato sensu postgraduate program, while 42 (25%) had not undertaken any specialization. Regarding the institution where they completed their master’s degree, a predominance of UFMG was observed, with 132 graduates, of whom 53 specifically indicated the UFMG School of Nursing. Other institutions were also mentioned, such as the Federal University of Paraíba, the State University of Paraíba, the Federal University of Alfenas, the Federal University of Juiz de Fora, the Federal University of Rio Grande do Norte, the Federal University of Viçosa, the State University of Southwest Bahia (UESB), the University of La Sabana (Colombia), the Federal University of São Paulo (UNIFESP), the Oswaldo Cruz Foundation (Fiocruz), and the University of São Paulo (USP).

During the master’s program, 38 graduates reported having received a scholarship, while 49 completed the program without this support. In the doctoral program, 49 graduates received a scholarship, whereas 32 completed the course without such assistance. Among the 87 master’s graduates from the 2017–2020 quadrennium who responded to the CPG questionnaire, 18 continued on to the doctoral program at EEUFMG, while five pursued doctoral studies at other institutions, including: the UFMG School of Medicine (n = 3), the UFMG School of Economic Sciences (n = 1), and Fiocruz (n = 1).

Before entering the PPGE/UFMG, most students already had diverse professional experiences, reflecting multiple forms of engagement in the fields of Nursing and Health. The data show that 59 graduates were already employed before beginning the graduate program, while another 39 started their professional activities during the course. After completing the master’s or doctoral degree, a progressive decline in new professional placements was observed: 28 within one year, 16 between one and three years, and only nine after three years.

Regarding participation in the labor market, the graduates indicate that most are employed in a single institution, with 168 respondents reporting only one workplace. A smaller group, composed of 33 graduates, works simultaneously in more than one institution, thus holding multiple employment contracts. Conversely, 17 graduates reported not being employed at the time of the questionnaire. Only three respondents indicated working independently.

Regarding the positions held by graduates, a marked difference is observed between those who completed the master’s degree and those who completed the doctorate. Among the 88 master’s graduates who responded to the questionnaire during the period analyzed, 43 reported working in care-related activities, suggesting that many graduates maintain direct involvement in health services. In addition, 44 reported holding supervisory or managerial roles, and three reported occupying coordination positions.

In contrast, among doctoral graduates, nine work in care, 13 hold supervisory or managerial roles, 17 occupy coordination positions, and teaching stands out as the main field of professional insertion, being mentioned by 64 respondents.

Beyond teaching, there is also an expansion in research and academic management activities among doctoral graduates. Among master’s graduates, 18 reported involvement in research, whereas among doctoral graduates this number was significantly higher, with 50 respondents working in the area.

In a total of 168 respondents, data on the occupation of coordination and director positions reveal that, among master’s graduates, four coordination roles and no director roles were identified, whereas among doctoral graduates, 17 coordination roles and one director position were reported. Of the 21 coordination roles mentioned by graduates, 17 are held by women and four by men.

Regarding employment arrangements, respondents indicated that most graduates (n = 128) work in public institutions, followed by the private sector, with 64 professionals, while only five graduates work independently. As for income, after completing the master’s degree, most graduates (n = 22) earn between four and five minimum wages, with incomes ranging from up to two to above nine minimum wages. Among doctoral graduates, the profile shifts: earnings above nine minimum wages predominate (n = 26), followed by ranges of six to seven (n = 17) and eight to nine minimum wages (n = 16), with no reports of earnings below two minimum wages.

Regarding participation in nursing representative organizations, the data reveal low engagement, with only 23 master’s graduates and nine doctoral graduates involved in professional entities.

## DISCUSSION

The analysis of the professional trajectories of PPGE/UFMG graduates, in light of Eliot Freidson’s sociology of professions, makes it possible to understand how stricto sensu training in Nursing contributes to the process of professionalization and to strengthening the status and autonomy of nurses within the professional field.

The age profile of PPGE/UFMG graduates resembles that found in other national studies on stricto sensu graduate education. Studies such as that by Gomes et al.^(13)^, which examined graduates of the Professional Master’s Program in Family Health (PROFSAÚDE), identified that 59.2% of respondents were up to 40 years old, while Heinzle et al.^(14)^ reported that 49.5% of graduates from the Master’s in Education at the Regional University of Blumenau (FURB) were between 31 and 40 years old figures that align with the findings from PPGE/UFMG.

The multidisciplinary nature of PPGE/UFMG highlights the program’s openness to integrating diverse forms of knowledge and professional practices, consolidating it as a space for the collective construction of knowledge. This characteristic aligns with global trends in health graduate education, marked by interprofessional training and interdisciplinary approaches^(15)^. Moreover, the heterogeneity of the student body and the multiple motivations for enrollment, as identified in recent studies, reinforce the importance of programs capable of welcoming and strengthening diverse academic and professional trajectories^(16)^.

From the perspective of the sociology of professions, interprofessional disputes and articulations have shaped the historical development of graduate education in Nursing in Brazil, strengthening its recognition as a field of knowledge^(3)^. By integrating professionals from different areas, PPGE/UFMG promotes the sharing of knowledge across professions and expands the production of technical and scientific expertise - an essential element for professional consolidation.

The data reveal a predominance of women in PPGE/UFMG, reflecting gender imbalances historically present in Nursing. International studies show low male participation, such as in the United States (9.6%), the Eastern Mediterranean (21%), Europe (16%), Southeast Asia (21%), and the Western Pacific (19%)^(17,18)^. In Brazil, between 1950 and 1999, only 2.37% of graduates from the University of São Paulo School of Nursing (EEUSP) were men^(19)^. Reviews indicate that the Nightingalean model, which strongly influenced Brazilian Nursing, reinforced stereotypes associating care with femininity, hindering men’s entry and permanence in the profession^(20)^.

A predominance of graduates from public higher education institutions is also observed, with participation from various public universities across the country, reinforcing the national relevance of PPGE/UFMG. The lower presence of graduates from private institutions may reflect inequalities in access and differences in educational trajectories that influence entry and persistence in graduate studies. According to Silva et al. (2023)^(16)^, motivations such as pursuing an academic career, professional qualification, and deepening knowledge are often associated with experiences in scientific initiation, research groups, and academic networks developed during undergraduate studies.

Public universities offer greater exposure to research and extension activities, whereas private institutions tend to prioritize practical training, which may influence preparation for graduate studies^(15)^. However, motivations for entering a graduate program do not always depend on the institution of origin, encompassing interests such as technical specialization, professional recognition, teaching, and academic identity. Thus, despite the predominance of public institutions in scientific production, students from diverse educational backgrounds view stricto sensu graduate education as an opportunity for professional and intellectual transformation^(21)^.

The data show that a large portion of former PPGE/UFMG students had already completed specialization courses before entering the stricto sensu graduate program, indicating a progressive educational trajectory. In another study conducted by Souza et al.^(22)^, which analyzed the profile of doctoral graduates in Nursing and Health from the State University of Southwest Bahia between 2014 and 2022, most doctoral students had also completed lato sensu programs beforehand. Thus, the PPGE/UFMG findings confirm that specialization continues to represent an important intermediate milestone in the qualification pathway for many professionals seeking to advance to stricto sensu training.

In light of Eliot Freidson’s sociology of professions, the high proportion of graduates with specialization degrees highlights one of the central pillars of his theory: credentialism, understood as the acquisition of formal titles and certifications as a means of legitimizing professional knowledge^(7)^. Freidsonian sociology identifies three fundamental pillars for recognizing an occupation as a profession^(23)^. Thus, the pursuit of specialization does not represent merely an individual effort toward qualification, but rather a collective movement of professional recognition, as demonstrated by PPGE graduates. In this context, titles – credentials - operate not only as markers of technical recognition and professional prestige but also influence the occupation of positions in the labor market and salary advancement.

Regarding fulltime dedication, the data indicate that this was not the first choice for most graduates. This scenario is consistent with findings from other studies, in which participants reported the need to maintain employment to support themselves and their families, intensifying workload and generating impacts on academic productivity, emotional wellbeing, and opportunities for broader involvement in research projects^(24)^. Thus, the situation identified at PPGE/UFMG is not isolated but reflects a condition observed in other institutions and experienced by many Nursing graduate students.

Most graduates entered the labor market before beginning the stricto sensu graduate program. This PPGE/UFMG finding aligns with studies conducted in Brazil^(25,26,27)^, which indicate that stricto sensu graduate programs serve as important mechanisms for consolidating and strengthening students’ professional trajectories. Similarly, in a crosssectional study conducted with doctoral students in Nursing in China, most participants already had prior professional experience and sought academic training as a means to expand opportunities in teaching and research, thereby consolidating their career plans^(28)^.

From a Freidsonian perspective, these data reinforce the idea that PPGE/UFMG functions as a space for professionalization within the Minas Gerais and Brazilian contexts, rather than as an entry point into the profession. One of the foundations of professionalism is the mastery of esoteric knowledge - knowledge not accessible to common sense - which grants technical authority and professional status^(22)^. In this sense, entering a stricto sensu graduate program after already being inserted in the labor market reflects a movement of credentialism, in which academic degrees are sought as instruments for expanding autonomy, prestige, and legitimacy within the field.

Regarding the positions held by graduates, a marked difference is observed between those who completed the master’s degree and those who completed the doctorate. Responses to the PPGE/UFMG questionnaire show that most master’s graduates continue to work in carerelated roles, whereas doctoral graduates are more concentrated in higher education and academic careers. Although the master’s degree deepens theoretical and practical training, this advancement does not always translate into institutional recognition or immediate career progression. Thus, care practice remains a central axis in the professional identity of master’s graduates, even in the face of expanded opportunities such as teaching. It is worth considering that, with the qualification obtained in the master’s program, these graduates may be better prepared for care delivery.

The doctorate, more than the master’s degree, has proven to be a powerful tool for insertion into academic life, reinforcing the role of stricto sensu graduate education as one of the main pathways to professionalization within the academic field of Nursing and other health areas.

Regarding the positions held by graduates, an important finding shows that although most PPGE/UFMG graduates are women, the proportion of men occupying management positions - particularly coordination roles - is comparatively higher. This reveals a gender imbalance in access to leadership positions, even within a field predominantly composed of women.

Nursing is widely viewed as a feminine profession because, as noted by Terra et al.^(29)^, this perception is strongly linked to the historical association of care with domestic labor. Other studies indicate that although nursing is a profession with a strong female presence, it is marked by unequal power relations, in which men - despite being a minority - more frequently occupy leadership positions and receive higher salaries^(29,30,31,32)^.

Gender disparity is a topic studied worldwide and is part of the Sustainable Development Goals (SDGs), a global initiative launched by the United Nations (UN) as part of the 2030 Agenda for Sustainable Development. Gender inequality is addressed in SDG 5. Although nursing is a predominantly female profession, it still exhibits gender inequalities in leadership spaces, as demonstrated by the PPGE/UFMG graduate data. This finding shows that the discourse of equity does not always translate into practice. Taminato et al.^(33)^ argue that a genuine commitment to the SDGs must promote real changes in the organizational culture of nursing, addressing the historical roots of this inequality.

Regarding remuneration, PPGE/UFMG data indicate that master’s graduates tend to have midrange earnings, whereas doctoral graduates show a more significant presence in higher education, research, and public management institutions - factors that positively influence salary levels. A survey by the Oswaldo Cruz Foundation (FIOCRUZ) supports these findings, showing that holding a doctoral degree is associated with greater employment stability and access to strategic positions, especially in public institutions, which contributes to higher salary ranges, as also evidenced in the PPGE data^(25)^.

According to the sociology of professions, this phenomenon strongly reflects credentialism, in which formal titles and certifications legitimize knowledge and differentiate individuals in the labor market^(7)^. The doctoral degree, as demonstrated by PPGE/UFMG graduates, expands access to more prestigious and betterpaid positions, such as teaching roles in public and private universities and academic management positions. Thus, the absence of doctoral graduates in the lowest income brackets reinforces the perspective of the sociology of professions, which argues that credentials reshape professional positions, strengthening the idea that scientificacademic knowledge is a driving force in the pursuit of status, prestige, and improved working conditions.

In light of the sociology of professions, professional associations are structuring elements of professionalization, as they legitimize knowledge, ensure autonomy in professional practice, and protect the interests of the category. They are essential for consolidating a profession as one of public trust^(7,34)^. These organizations work on behalf of nursing and society, advocating for political projects of training and qualification that address both the interests of nursing professionals and broader social demands. They represent professionals across the various fields in which they are employed^(34)^.

The participation of PPGE/UFMG graduates in nursing representative organizations reveals a pattern of low engagement. This limited number of responses regarding involvement with civil entities of the profession is a concerning finding, given that, as discussed by Carregal et al.^(3)^, stricto sensu graduate programs are considered privileged spaces for critical production and for strengthening professional identity, aligning with Freidsonian conceptions of professionalization.

## CONCLUSION

From the perspective of the sociology of professions, stricto sensu graduate education reinforces the pursuit of a distinct body of knowledge - also referred to by Freidson as esoteric knowledge - which legitimizes the technical authority of graduates in their fields of work. This authority is accompanied by professional autonomy, particularly in the ability to lead research projects, conduct educational processes, and intervene in institutional spaces, as demonstrated by several graduates.

Despite the educational advances and academic achievements promoted by PPGE/UFMG, graduates also reveal important challenges faced during the training process and even after completing the program, which shape their professional trajectories. Gender inequalities were observed, as well as low adherence to scholarship programs. Although women represent the majority of participants, they proportionally occupy fewer management positions compared with men, corroborating findings from other studies.

The expertise obtained through master’s and doctoral credentials not only strengthens Nursing as a scientific field but also expands its areas of practice, placing graduates in key spaces of decisionmaking, management, research, and public policy. However, there is limited awareness regarding participation in professional associations—an important dimension of professionalization in Freidson’s framework - which calls for promotional actions on the part of graduate programs.

As with any study, this research presents limitations that must be acknowledged, particularly the portion of graduates who did not respond to the questionnaire sent by the program committee. Therefore, further studies are needed to follow graduates’ trajectories over time, broadening the understanding of the impacts of stricto sensu training at different stages of professional development.

Based on the findings of this study, the relevance of PPGE/UFMG is reaffirmed as a training space for social actors with distinct and critical knowledge, committed to science, teaching, and society. The profile of its graduates reveals not only the individual impacts of academic training but also the institutional and collective effects of a program that has consolidated itself as a national reference, as evidenced by its rise to excellence.

## Data Availability

The entire dataset supporting the results of this study was published in the article itself.
