# Supplementary material for: Profile of graduates from the UFMG Nursing Graduate Program (2017–2020): a Freidsonian analysis
Source: Rev Esc Enferm USP. 2026 Jul 24;60:e20260234. doi: 10.1590/1980-220X-REEUSP-2026-0234en (PMC13419338; doi:10.1590/1980-220X-REEUSP-2026-0234en)
Supplement: Translation

# Translation: Perfil dos egressos da pós-graduação em Enfermagem da UFMG (2017–2020): análise freidsoniana

**DOI:** 10.1590/1980-220X-REEUSP-2026-0234pt

**Authors:** Bárbara Costa Moreira, Pacita Geovana Gama de Sousa Aperibense, Maria Itayra Padilha, Camila Pureza Guimarães da Silva, Maria Angélica de Almeida Peres, Fernanda Batista Oliveira Santos

**Affiliations:** 1 Universidade Federal de Minas Gerais, Escola de Enfermagem, Programa de Pós-Graduação em Enfermagem, Belo Horizonte, MG, Brasil.; 2 Universidade Federal do Rio de Janeiro, Departamento de Enfermagem Fundamental, Macaé, RJ, Brasil.; 3 Universidade Federal de Santa Catarina, Departamento de Enfermagem e do Programa de Pós-graduação em Enfermagem, Florianópolis, SC, Brasil.; 4 Universidade Federal do Rio de Janeiro, Escola de Enfermagem Anna Nery, Departamento de Enfermagem Fundamental, RJ, Brasil.

**Keywords:** Educação de Pós-Graduação, Programas de Pós-Graduação em Saúde, Educação em Enfermagem, Análise Institucional, Avaliação Educacional

## Abstract

**Objetivo:**

Caracterizar o perfil demográfico, acadêmico e profissional dos egressos da pós-graduação *stricto sensu* em Enfermagem da Escola de Enfermagem da Universidade Federal de Minas Gerais no quadriênio de 2017 a 2020 à luz da sociologia das profissões de Eliot Freidson.

**Método:**

Estudo qualitativo histórico-social, conduzido segundo o *Consolidated criteria for reporting qualitative research* (COREQ). Os dados foram coletados entre outubro de 2024 e abril de 2025 no banco de egressos do Programa e na Plataforma Lattes, analisados por crítica interna e externa segundo pertinência, representatividade e exaustividade, e interpretados à luz da Sociologia das Profissões de Eliot Freidson.

**Resultados:**

A análise freidsoniana evidenciou credencialismo, fortalecimento do *status* e autonomia profissional, maior inserção em posições de prestígio entre doutores, persistindo desigualdades de gênero em liderança e diferenças salariais.

**Conclusão:**

A pesquisa reafirma a relevância do Programa de pós-graduação em enfermagem mineiro como espaço formador de profissionais críticos e comprometidos com a ciência, a docência e a sociedade. O perfil dos egressos evidenciou impactos individuais e institucionais, consolidando o programa como referência nacional em excelência acadêmica.

## INTRODUÇÃO

As intensas transformações industriais, econômicas e políticas ocorridas no Brasil na década de 1960 determinaram a promulgação da Reforma Universitária de 1968, repercutindo mudanças nos cursos de graduação e a criação de Programas de Pós-graduação^(1,2,3)^. Na Enfermagem, a consolidação da pós-graduação stricto sensu seguiu um percurso não linear, marcado pelo fortalecimento da profissão, ampliação da produção científica, consolidação nacional e internacional dos programas e contribuição para a internacionalização da ciência brasileira^(4)^.

Programas de Pós-Graduação são constituídos por corpos docente, técnico-administrativo e discente, cuja dinâmica envolve múltiplos olhares e experiências. A visão e a missão desses programas exigem de seus atores sociais uma reflexão assistencial e gerencial voltada ao desenvolvimento da ciência e da tecnologia na área da Saúde. Portanto, considerar o desenvolvimento desses sujeitos torna-se essencial para compreender os impactos sociais, políticos e culturais gerados, subsidiando o aprimoramento e o fortalecimento dos programas^(4)^.

Em Minas Gerais, a Escola de Enfermagem da Universidade Federal de Minas Gerais (EEUFMG) tem, desde sua fundação em 1933, desempenhado um papel central na profissionalização da enfermagem. Ela foi responsável pela criação do primeiro Programa de Pós-Graduação em Enfermagem *Stricto Sensu* (PPGE) no estado, sendo o mesmo instituído em 1993 com a primeira turma de mestrado. O doutorado ocorreu em 2004, ampliando ainda mais sua contribuição para a formação de pesquisadores e para o avanço científico da enfermagem brasileira^(3)^.

O fortalecimento da pós-graduação no Brasil é evidenciado a partir de 1981, com a criação do Plano Nacional de Pós-Graduação *Stricto Sensu* pela Coordenação de Aperfeiçoamento de Pessoal de Nível Superior (CAPES). Sendo responsável atualmente pela avaliação quadrienal dos programas, a instituição recomenda que, no que diz respeito à formação, se considere a trajetória dos egressos, analisando sua inserção profissional e os impactos sociais, políticos e econômicos advindos de sua capacitação^(5,6)^.

Desde 2020, o PPGE/UFMG realiza o acompanhamento de seus egressos após a defesa e pelos cinco anos seguintes à conclusão do curso, conforme recomendado pela CAPES. Os egressos são direcionados ao site do programa para preencher um formulário contendo informações sobre dados de identificação, titulação, formação e atuação profissional no período da coleta.

À luz da sociologia das profissões é possível compreender a consolidação da pós-graduação em enfermagem como parte de um processo mais amplo de profissionalização. Segundo Freidson, uma profissão se estrutura a partir de três pilares fundamentais: a expertise, a autonomia e o credencialismo^(7)^. Neste sentido, os PPGEs tornam-se espaços importantes para a constituição de um corpo de conhecimento técnico-científico, contribuindo para a consolidação da enfermagem como campo profissional autônomo e reconhecido. A identificação da trajetória profissional dos egressos, portanto, é fundamental para avaliar como essa formação repercute em sua inserção no mercado de trabalho, em seu prestígio e em sua autoridade técnica, refletindo os mecanismos de reprodução e valorização da profissão no contexto brasileiro contemporâneo.

Apesar dos avanços da pós-graduação *stricto sensu* em Enfermagem no Brasil, ainda são escassos os estudos que analisam criticamente os impactos dessa formação nas trajetórias profissionais dos egressos^(8)^. A importância de investigações nessa direção é reforçada pela Agenda de Prioridades de Pesquisa do Ministério da Saúde (APPMS), que destaca a necessidade de avaliar o impacto das ofertas educacionais na qualificação dos profissionais do Sistema Único de Saúde (SUS)^(9)^. De forma complementar, o documento “Prioridades em Pesquisa para Enfermagem: Proposta Preliminar” da Associação Brasileira de Enfermagem (ABEn) aponta a relevância de estudos sobre a formação stricto sensu, sua capacidade de desenvolver competências, qualificar o trabalho em saúde e contribuir para a gestão do SUS^(10)^.

Nesse contexto, a compreensão das trajetórias profissionais dos egressos do PPGE/UFMG no quadriênio 2017–2020 torna-se essencial para subsidiar discussões sobre a valorização da Enfermagem e o fortalecimento do sistema público de saúde. Diante disso, questiona-se: como tem se delineado a trajetória profissional dos egressos do PPGE/UFMG, no quadriênio 2017 a 2020, após a conclusão de seus mestrados e/ou doutorados?

O presente estudo tem como objetivo caracterizar o perfil demográfico, acadêmico e profissional dos egressos da pós-graduação *stricto sensu* em Enfermagem da Escola de Enfermagem da Universidade Federal de Minas Gerais no quadriênio de 2017 a 2020, à luz da sociologia das profissões de Eliot Freidson.

Parte-se do pressuposto de que a titulação *stricto sensu* em enfermagem representa mais do que uma qualificação acadêmica. Ela atua como mecanismo de diferenciação no campo profissional, conferindo autoridade técnica e *status* aos egressos. À luz da teoria da sociologia das profissões, supõe-se que a pós-graduação contribui para a construção de um saber especializado, legitima posições de prestígio no mercado de trabalho e reforça processos de profissionalização da enfermagem.

## MÉTODO

### Tipo de Estudo

O presente estudo insere-se no campo da História, com uma dimensão sócio-histórica, voltada para o exame das especificidades de uma sociedade, incluindo os modos e mecanismos de organização social, as relações entre grupos, instituições e indivíduos, bem como os processos de transformação social^(11,12)^. A pesquisa está alinhada aos domínios da história da enfermagem. A abordagem adotada é qualitativa utilizando-se a História Textual, com análise documental de fontes escritas.

O estudo foi conduzido seguindo as recomendações da diretriz *Consolidated criteria for reporting qualitative research* COREQ para pesquisas qualitativas.

### Local

O estudo foi realizado na Escola de Enfermagem da Universidade Federal de Minas Gerais (EEUFMG), em Belo Horizonte, que sedia o Programa de Pós-Graduação em Enfermagem (PPGE). A delimitação temporal e espacial abrange o quadriênio de 2017 a 2020, período marcado por um contexto de liberalismo econômico, ataques à democracia e às instâncias de participação social. Soma-se a esse cenário a pandemia de COVID-19, que exigiu reestruturações nos setores da saúde e da educação.

### Fontes e Coleta de Dados

As fontes textuais que compuseram este estudo foram obtidas junto ao Colegiado de Pós-Graduação (CPG) da Escola de Enfermagem da UFMG, incluindo o banco de dados de egressos do PPGE/UFMG. Buscas na Plataforma Lattes, mantida pelo Conselho Nacional de Desenvolvimento Científico e Tecnológico (CNPq), foram realizadas para compor dados sobre a trajetória profissional de egressos. A escolha dessa plataforma justifica-se por ser o principal sistema virtual brasileiro de currículos científicos e por ter acesso aberto, permitindo assim a confirmação da identidade dos egressos.

Foram incluídas como fontes textuais aquelas que estavam atualizadas e vinculadas ao nome completo do egresso. Foram excluídos registros incompletos, desatualizados ou sem correspondência verificável com o banco de dados do programa.

Os dados foram coletados pela pesquisadora principal entre os meses de outubro de 2024 e abril de 2025.

### Análise e Tratamento dos Dados

O tratamento das fontes incluiu a composição e organização de um banco na plataforma Google Drive, descritos e organizados por ano (2017 a 2020). Uma vez catalogadas, as fontes foram categorizadas a partir de critérios metodológicos estabelecidos como pertinência das fontes, suficiência, representatividade, exaustividade e homogeneidade. As fontes textuais foram analisadas criticamente quanto à veracidade e relevância, por meio de crítica interna e externa^(12)^. Os achados foram analisados à luz da Sociologia das Profissões de Eliot Freidson.

### Aspectos Éticos

A pesquisa foi apreciada pelo Comitê de Ética em Pesquisa da UFMG, garantindo o assentimento e a adequação às normativas éticas e legais vigentes. Foi aprovada sob o parecer n. 7.115.336 no ano de 2024.

## RESULTADOS

No quadriênio de 2017 a 2020, o Programa de Pós-Graduação em Enfermagem da Universidade Federal de Minas Gerais (PPGE/UFMG) titulou 218 discentes, sendo 130 mestres e 88 doutores. Do total de egressos, 168 responderam ao questionário, o que representam 77,06% do total; destes, 87 eram mestres e 81 doutores.

Do total de egressos pertencentes a esse quadriênio, 190 eram mulheres e 28 homens, o que evidencia uma expressiva predominância feminina (87%). A faixa etária dos egressos do PPGE/UFMG está presente no gráfico abaixo (Figura 1), mostrando uma maior ocorrência aos 36 anos, com 23 egressos, seguido pelas idades de 37 (n = 18), 38 (n = 13) e 34 (n = 14) anos.

**Figura 1:** Faixa etária dos egressos do PPGE.

Fonte: Relatório De Análise Estatística Dos Egressos Da Pós-Graduação (2017–2020), 2025.

A formação inicial dos egressos do PPGE/UFMG é majoritariamente composta por enfermeiros, somando 147 profissionais. O programa também acolheu, ao longo do quadriênio 2017–2020, um profissional de Educação Física, um de Estatística, dois farmacêuticos, três gestores de saúde e oito nutricionistas, um pedagogo, quatro psicólogos e um terapeuta ocupacional.

Após identificar a predominância de enfermeiros entre os egressos, observa-se, nesse grupo, uma expressiva participação feminina. Os dados do PPGE/UFMG indicam que, no período de 2017 a 2020, 128 mulheres concluíram o curso, em contraste com apenas 19 homens.

Em relação ao local de formação dos egressos, 62 estudantes graduaram-se pela UFMG, 63 em outras universidades públicas do Brasil e 36 em instituições de nível superior privadas.

Dos 168 egressos que responderam ao questionário do CPG, 126 (75%) possuíam pós-graduação *lato sensu*, enquanto 42 (25%) não haviam realizado especialização alguma. Acerca do local de formação do mestrado, observou-se uma predominância da UFMG, com 132 egressos, dos quais 53 indicaram especificamente a Escola de Enfermagem da UFMG. Outros locais também foram citados, como a Universidade Federal da Paraíba, a Universidade Estadual da Paraíba, a Universidade Federal de Alfenas, Universidade Federal de Juiz de Fora, a Universidade Federal do Rio Grande do Norte, Universidade Federal de Viçosa, Universidade Estadual do Sudoeste da Bahia (UESB), a Universidade de la Sabana - Colômbia, a Universidade Federal de São Paulo (UNIFESP), Fundação Oswaldo Cruz (Fiocruz), e a Universidade de São Paulo (USP).

Durante o mestrado, 38 egressos relataram ter recebido bolsa de estudos, enquanto 49 cursaram o programa sem esse apoio. No doutorado, 49 egressos foram beneficiados com bolsa, enquanto 32 realizaram o curso sem o recebimento desse auxílio. Dos 87 mestrandos egressos do quadriênio 2017–2020 que responderam ao questionário do CPG, 18 prosseguiram para o doutorado na EEUFMG enquanto cinco seguiram para outras instituições como: Faculdade Medicina UFMG (n = 3), Faculdades de Ciências Econômicas da UFMG (n = 1) e Fiocruz (n = 1).

Antes de ingressarem no PPGE/UFMG, a maioria dos estudantes já possuía experiências profissionais diversas, refletindo múltiplas inserções no campo da Enfermagem e da Saúde. Os dados mostram que 59 egressos já estavam inseridos no mercado de trabalho antes da pós-graduação, enquanto outros 39 iniciaram suas atividades profissionais durante o curso. Após a conclusão do mestrado ou doutorado, observa-se uma redução progressiva em novas inserções profissionais: 28 até um ano depois, 16 entre um e três anos, e apenas nove após três anos.

Sobre a adesão ao mercado de trabalho, os egressos demonstram que a maioria está atuando em uma única instituição, com 168 respondentes. Um grupo menor, composto por 33 egressos, atua simultaneamente em mais de uma instituição, configurando mais de um vínculo empregatício. Em contrapartida, 17 egressos declararam não estar trabalhando no momento da resposta ao questionário. Apenas três respondentes indicaram atuar de forma autônoma.

Acerca dos cargos ocupados pelos egressos, observa-se uma diferença marcante entre aqueles que concluíram o mestrado e os que finalizaram o doutorado. Dos 88 mestres que responderam ao questionário no período analisado, 43 relataram atuar em atividades assistenciais, o que sugere que muitos egressos mantêm uma atuação direta nos serviços de saúde. Além deste dado, 44 relataram uma relação com supervisão e gerência, e três em cargos de coordenação.

Em contraste, no caso dos doutores, nove egressos atuam na assistência, 13 exercem funções de supervisão e gerência, 17 atuam em cargos de coordenação, enquanto a docência se destaca como o principal campo de inserção profissional, sendo mencionada por 64 colaboradores do estudo.

Além da docência, observa-se também uma ampliação da inserção em atividades de pesquisa e gestão acadêmica entre os egressos do doutorado. Dos participantes do mestrado, 18 declararam envolvimento com pesquisas, enquanto no doutorado esse número foi significativamente maior, com 50 respondentes atuando na área.

Em um universo de 168 respondentes, os dados sobre ocupação de cargos de coordenação e diretoria revelam que, entre os egressos do mestrado, foram identificadas quatro coordenações e nenhuma diretoria, enquanto no doutorado registraram-se 17 coordenações e uma diretoria. Das 21 coordenações relatadas pelos egressos, 17 são ocupadas por mulheres e quatro por homens.

Quanto aos regine de vínculo empregatícios dos egressos, os respondentes mostraram que em sua maioria (n = 128) trabalham em instituições públicas, seguido pelo setor privado, com 64 profissionais, enquanto apenas cinco egressos atuam de forma autônoma. E quanto a seus rendimentos, após o mestrado, a maioria dos egressos (n = 22) recebe entre quatro e cinco salários-mínimos, com rendas distribuídas de até dois a acima de nove salários. No doutorado, o perfil muda: prevalecem rendas acima de nove salários-mínimos (n = 26), seguidas por faixas de seis a sete (n = 17) e oito a nove salários-mínimos (n = 16), sem registros abaixo de dois salários.

Quanto a participação dos egressos do PPGE/UFMG em entidades representativas da enfermagem, revela-se um movimento de baixa adesão, com apenas 23 mestres e nove doutores envolvidos em organizações da profissão.

## DISCUSSÃO

A análise do perfil da trajetória profissional dos egressos do PPGE/UFMG, à luz da sociologia das profissões de Eliot Freidson, possibilita compreender como a formação *stricto sensu* em Enfermagem contribui para o processo de profissionalização e para o fortalecimento do status e da autonomia do enfermeiro no campo profissional.

A idade dos egressos do PPGE/UFMG demonstra um perfil semelhante ao encontrado em outras pesquisas nacionais sobre pós-graduação *stricto sensu*. Estudos como o de Gomes e colaboradores^(13)^, que apresenta pesquisa com egressos do Mestrado Profissional em Saúde da Família (PROFSAÚDE), identificaram que 59,2% dos respondentes tinham até 40 anos, enquanto Heinzle e colaboradores^(14)^ apontaram que 49,5% dos egressos do Mestrado em Educação da Universidade Regional de Blumenau (FURB) estavam entre 31 e 40 anos - dados que se assemelham aos achados do PPGE/UFMG.

A característica multidisciplinar do PPGE/UFMG evidencia a abertura do programa para a integração de diferentes saberes e práticas profissionais, consolidando-o como um espaço de construção coletiva do conhecimento. Essa característica acompanha as tendências da pós-graduação em saúde global, marcada por formações interprofissionais e abordagens interdisciplinares^(15)^. Além disso, a heterogeneidade do corpo discente e as múltiplas motivações para o ingresso, apontadas por estudos recentes, reforçam a importância de programas capazes de acolher e potencializar distintas trajetórias acadêmicas e profissionais^(16)^.

Na perspectiva da sociologia das profissões, disputas e articulações interprofissionais marcaram a historicidade da pós-graduação em Enfermagem no Brasil, fortalecendo seu reconhecimento como campo de conhecimento^(3)^. Ao integrar profissionais de diversas áreas, o PPGE/UFMG promove o compartilhamento entre profissões e amplia a produção de saber técnico e científico, elemento central para a consolidação profissional.

Os dados evidenciam a predominância feminina no PPGE/UFMG, refletindo desequilíbrio de gênero historicamente presentes na Enfermagem. Estudos internacionais apontam baixa participação masculina, como nos EUA (9,6%), no Mediterrâneo Oriental (21%), na Europa (16%), no Sudeste Asiático (21%) e no Pacífico Ocidental (19%)^(17,18)^. No Brasil, entre 1950 e 1999, apenas 2,37% dos egressos da Escola de Enfermagem da Universidade de São Paulo (EEUSP) eram homens^(19)^. Revisões indicam que o modelo nightingaleano, com forte influência na Enfermagem brasileira, reforçou estereótipos que associam o cuidado ao feminino, dificultando a escolha e permanência masculina na profissão^(20)^.

Observa-se a predominância de vínculos com instituições públicas de ensino, com participação de egressos de diversas universidades públicas do país, reforçando a relevância nacional do PPGE/UFMG. A baixa presença de titulados em faculdades privadas pode refletir desigualdades de acesso e diferenças na trajetória formativa, que influenciam o ingresso e a permanência na pós-graduação. Segundo Silva et al. (2023)^(16)^, motivações como carreira acadêmica, qualificação e aprofundamento de conhecimentos estão frequentemente associadas a experiências de iniciação científica, grupos de pesquisa e redes acadêmicas vivenciadas na graduação.

As universidades públicas oferecem maior exposição à pesquisa e extensão, enquanto faculdades privadas tendem a priorizar a formação prática, o que pode impactar a preparação para a pós-graduação^(15)^. Contudo, motivações para o ingresso nem sempre dependem da instituição de origem, abrangendo interesses como especialização técnica, reconhecimento profissional, docência e identidade acadêmica. Assim, apesar da predominância pública na produção científica, estudantes de diferentes formações veem na pós-graduação stricto sensu uma oportunidade de transformação profissional e intelectual^(21)^.

Os dados identificaram que grande parte dos ex-discentes do programa já havia cursado especializações antes de ingressar na pós-graduação stricto sensu, caracterizando uma trajetória formativa progressiva. Em uma outra pesquisa, realizada por Souza e colaboradores^(22)^, ao analisarem o perfil dos egressos do doutorado em Enfermagem e Saúde da Universidade Estadual do Sudoeste da Bahia entre os anos de 2014 e 2022, observou-se que a maioria dos doutorandos também havia realizado cursos *lato sensu* previamente. Assim, os dados do PPGE/UFMG confirmam que a especialização ainda representa um importante marco intermediário no percurso de qualificação de muitos profissionais que buscam avançar para o *stricto sensu*.

À luz da sociologia das profissões de Eliot Freidson, a elevada proporção de egressos com especializações evidencia um dos pilares centrais de sua teoria: o credencialismo, entendido como a aquisição dos títulos e certificações formais como forma de legitimação do saber profissional^(7)^. A sociologia freidsoniana destaca três pilares fundamentais para o reconhecimento de uma ocupação como profissão^(23)^. Desta forma, a busca por especializações não representa apenas um esforço individual por qualificação, mas sim um movimento coletivo de reconhecimento profissional como demonstrado pelos egressos do PPGE, no qual os títulos, ou seja, as credenciais, operam como formas de reconhecimento técnico e prestígio profissional, mas também influencia na ocupação de cargos no mercado de trabalho e melhoria salarial.

No que se refere à dedicação exclusiva, os dados indicam que essa não foi a primeira escolha para a maioria dos egressos. Esse cenário é visto em outros estudos, em que os participantes da pesquisa trouxeram a necessidade de manter vínculos empregatícios para garantir o sustento pessoal e familiar intensificando a sobrecarga, gerando impactos na produtividade acadêmica, no bem-estar emocional, e nas possibilidades de inserção mais ampla em projetos de pesquisa^(24)^. Assim, o quadro identificado no PPGE/UFMG não é isolado, mas reflete uma condição vista em outra instituições, enfrentada por muitos pós-graduandos na Enfermagem.

A inserção profissional dos egressos ocorreu em sua maioria antes da entrada na pós-graduação *stricto sensu*. Esse dado do PPGE/UFMG está em consonância com estudos realizados no Brasil^(25,26,27)^, que apontam os cursos de pós-graduação stricto sensu como importantes mecanismos de consolidação e qualificação das trajetórias profissionais dos discentes. De modo semelhante, em estudo transversal realizado com estudantes de doutorado em enfermagem na China, identificou-se que a maioria dos estudantes já possuía inserção profissional anterior e buscava, por meio da formação acadêmica, ampliar suas oportunidades nas áreas de ensino e pesquisa, consolidando assim seus projetos de carreira^(28)^.

Sob o olhar freidsoniano, esses dados reforçam a ideia de que o PPGE/UFMG atua como um espaço de profissionalização no cenário mineiro e brasileiro mais do que como uma porta de entrada para o exercício profissional. Uma das bases do profissionalismo está no domínio de um conhecimento esotérico, não acessível ao senso comum, que confere autoridade técnica e status profissional^(22)^. Nesse sentido, a entrada na pós-graduação *stricto sensu* por atores sociais já inseridos no mercado demonstra um movimento de credencialismo, em que a titulação acadêmica é buscada como instrumento de ampliação da autonomia, do prestígio e da legitimidade no campo de atuação.

Acerca dos cargos ocupados pelos egressos, observa-se uma diferença marcante entre aqueles que concluíram o mestrado e os que finalizaram o doutorado. As respostas ao questionário do PPGE/UFMG mostram que a maioria dos mestres permanece atuando em funções assistenciais, enquanto os doutores se concentram mais no ensino superior e na carreira acadêmica. Embora o título de mestre aprofunde a formação teórica e prática, esse avanço nem sempre se traduz em reconhecimento institucional ou progressão imediata na carreira. Assim, a prática assistencial permanece como eixo central na identidade profissional dos egressos do mestrado, mesmo diante da ampliação de oportunidades, como a docência. Vale refletir que, provavelmente, com qualificação no mestrado, este egresso esteja mais preparado para o cuidado assistencial. Já o doutorado, mais do que o mestrado, tem se mostrado uma ferramenta potente de inserção na vida docente, reforçando o papel da pós-graduação stricto sensu como um dos principais caminhos de profissionalização no campo acadêmico da Enfermagem e de outras áreas da saúde.

Sobre os cargos ocupados pelos egressos, um achado importante demonstra que maioria dos egressos do PPGE/UFMG são mulheres, porém, a aquisição dos cargos de gestão está proporcionalmente maior para os homens nas coordenações. Esse fato demostra uma desigualdade de gênero no acesso a cargos de liderança, mesmo em um campo composto em sua maioria pelo feminino.

A enfermagem é vista como uma profissão feminina, pois, segundo Terra et al.^(29)^, esta visão é muito ligada ao contexto histórico de que o cuidado é tido como extensão do trabalho doméstico. Outros estudos apontam que embora a enfermagem seja uma profissão com maior presença feminina, ela é marcada por relações desiguais de poder, onde os homens, mesmo sendo uma minoria, ocupam com maior frequência cargos de chefia e recebem salários superiores^(29,30,31,32)^.

A disparidade de gênero é uma temática estudada mundialmente e faz parte dos Objetivos de Desenvolvimento Sustentável (ODS), que é uma iniciativa global lançada pela Organização das Nações Unidas (ONU) como parte da Agenda 2030 para o Desenvolvimento Sustentável. A desigualdade de gênero está abordada na ODS 05. Por mais que a enfermagem seja uma profissão majoritariamente feminina, ela ainda apresenta desigualdades de gênero em seus espaços de liderança, conforme demonstrado pelos dados dos egressos do PPGE/UFMG. Tal dado revela que o discurso da equidade nem sempre se reflete na prática. Taminato et al.^(33)^ demonstram que é essencial que o compromisso com os ODS promova mudanças reais na cultura organizacional da enfermagem, enfrentando as raízes históricas dessa desigualdade.

Acerca da remuneração, os dados do PPGE/UFMG indicam que os mestres apresentam rendimentos médios, enquanto os doutores mostram inserção mais expressiva em instituições de ensino superior, pesquisa e gestão pública, o que se reflete positivamente nos salários. Levantamento da Fundação Oswaldo Cruz (FIOCRUZ) corrobora esses achados, apontando que o título de doutor está associado a maior estabilidade empregatícia e à ocupação de cargos estratégicos, especialmente em instituições públicas, fatores que contribuem para faixas salariais mais elevadas, tal como evidenciado nos dados do PPGE^(25)^.

Segundo a sociologia das profissões, esse evento diz muito sobre o credencialismo, onde os títulos e certificações formais legitimam o conhecimento e diferenciam os indivíduos no mercado de trabalho^(7)^. O título de doutor, como demonstrado pelos egressos do PPGE/UFMG, amplia o acesso a posições mais prestigiadas e bem remuneradas, como cargos de docência em universidades públicas e privadas e gestão acadêmica. Assim, a ausência de egressos do doutorado nas faixas de renda mais baixas corrobora a perspectiva da sociologia das profissões, que indica que as credenciais redefinem as posições profissionais, fortalecendo o argumento de que o saber científico-acadêmico é uma mola propulsora na busca por status, prestígio e melhores condições de trabalho.

À luz da sociologia das profissões, as entidades de classe são estruturantes para a profissionalização, pois elas legitimam o conhecimento, asseguram a autonomia do exercício profissional e protegem os interesses da categoria, sendo essenciais para consolidar uma profissão como de consulta^(7,34)^. Elas trabalham para a enfermagem e para a sociedade e defendem projetos políticos de formação e qualificação que levem aos interesses dos profissionais da enfermagem e às demandas sociais. Elas representam os profissionais nos diversos campos de atuação em que estão inseridos^(34)^.

A participação dos egressos do PPGE/UFMG em entidades representativas da enfermagem revela um movimento de baixa adesão. Esse baixo quantitativo de respostas referentes ao envolvimento dos egressos do PPGE/UFMG com as entidades civis de sua classe profissional é um dado de alerta visto que, como abordado por Carregal et al.^(3)^, a pós-graduação *stricto sensu* é tida como um espaço privilegiado de produção crítica e de fortalecimento da identidade profissional, alinhando-se às concepções freidsonianas de profissionalização.

## CONCLUSÃO

A pós-graduação *stricto sensu* reforçou a busca por um conhecimento próprio, também chamado de isotérico, como abordado por Freidson, que legitima a autoridade técnica dos egressos em seus campos de trabalho. Essa autoridade vem junto à autonomia profissional, sobretudo na capacidade de tomar frente a projetos de pesquisa, conduzir processos formativos e intervir nos espaços institucionais assim como demostrado por diversos egressos.

Apesar dos avanços formativos e das conquistas acadêmicas promovidas pelo PPGE/UFMG, os egressos também revelam importantes desafios enfrentados no processo de formação, e até mesmo após o término do curso, que culminam em sua trajetória profissional. Foram observadas tanto desigualdades de gênero, baixa adesão a bolsas de estudos e embora as mulheres representem a maioria entre os participantes da pesquisa, elas ocupam proporcionalmente menos cargos de gestão em comparação aos homens, corroborando outros estudos.

A expertise obtida por meio da credencial do mestrado e doutorado, não apenas fortalece o campo da Enfermagem como ciência, mas também aumenta seus campos de atuação, inserindo seus egressos em espaços importantes de decisão, gestão, pesquisa e políticas públicas. Contudo, há uma baixa conscientização em relação à participação associativa, ponto importante da profissionalização na lógica freidsoniana, que merece ações promotoras por parte dos PPG.

Como todo estudo, esta pesquisa apresenta limitações que devem ser reconhecidas, com destaque para a parcela de egressos que não respondeu ao questionário encaminhado pelo colegiado. Dessa forma, torna-se necessária a realização de novos estudos que acompanhem a trajetória dos egressos ao longo do tempo, ampliando a compreensão acerca dos impactos da formação *stricto sensu* em diferentes etapas da trajetória profissional.

A partir dos achados desta pesquisa, reafirma-se a relevância do PPGE/UFMG como espaço formador de atores sociais com conhecimento próprios e críticos, comprometidos com a ciência, docência e sociedade. O perfil dos egressos revela não apenas os impactos individuais da formação acadêmica, mas também os efeitos institucionais e coletivos do programa que se consolida como referência nacional, evidenciado pela sua ascensão à excelência.

## Data Availability

Todo o conjunto de dados que dá suporte aos resultados deste estudo foi publicado no próprio artigo.

# Translation: Perfil de los egresados del posgrado en Enfermería de la UFMG (2017–2020): un análisis freidsoniano

**DOI:** 10.1590/1980-220X-REEUSP-2026-0234es

**Authors:** Bárbara Costa Moreira, Pacita Geovana Gama de Sousa Aperibense, Maria Itayra Padilha, Camila Pureza Guimarães da Silva, Maria Angélica de Almeida Peres, Fernanda Batista Oliveira Santos

**Affiliations:** 1 Universidade Federal de Minas Gerais, Escola de Enfermagem, Programa de Pós-Graduação em Enfermagem, Belo Horizonte, MG, Brasil.; 2 Universidade Federal do Rio de Janeiro, Departamento de Enfermagem Fundamental, Macaé, RJ, Brasil.; 3 Universidade Federal de Santa Catarina, Departamento de Enfermagem e do Programa de Pós-graduação em Enfermagem, Florianópolis, SC, Brasil.; 4 Universidade Federal do Rio de Janeiro, Escola de Enfermagem Anna Nery, Departamento de Enfermagem Fundamental, RJ, Brasil.

**Keywords:** Educación de Posgrado, Programas de Posgrado en Salud, Educación en Enfermería, Análisis Institucional, Evaluación Educacional

## Abstract

**Objetivo:**

Caracterizar el perfil demográfico, académico y profesional de los egresados del programa de posgrado stricto sensu en Enfermería de la Escuela de Enfermería de la Universidad Federal de Minas Gerais durante el cuatrienio 2017–2020, a la luz de la sociología de las profesiones de Eliot Freidson.

**Método:**

Estudio cualitativo histórico-social, conducido según las directrices COREQ. Los datos fueron recolectados entre octubre de 2024 y abril de 2025 en la base de egresados del Programa y en la Plataforma Lattes, analizados mediante crítica interna y externa según pertinencia, representatividad y exhaustividad, e interpretados a la luz de la Sociología de las Profesiones de Eliot Freidson.

**Resultados:**

El análisis freidsoniano evidenció credencialismo, fortalecimiento del estatus y de la autonomía profesional, y una mayor inserción de doctores en posiciones de prestigio, persistiendo desigualdades de género en liderazgo y diferencias salariales.

**Conclusión:**

La investigación reafirma la relevancia del programa de posgrado en Enfermería de Minas Gerais como un espacio formador de profesionales críticos y comprometidos con la ciencia, la docencia y la sociedad. El perfil de los egresados evidenció impactos individuales e institucionales, consolidando el programa como referencia nacional en excelencia académica.

## INTRODUCCIÓN

Las intensas transformaciones industriales, económicas y políticas que tuvieron lugar en Brasil en la década de 1960 determinaron la promulgación de la Reforma Universitaria de 1968, lo que repercutió en cambios en los programas de pregrado y en la creación de programas de posgrado^(1,2,3)^. En Enfermería, la consolidación de la posgrado stricto sensu siguió un recorrido no lineal, marcado por el fortalecimiento de la profesión, la ampliación de la producción científica, la consolidación nacional e internacional de los programas y la contribución a la internacionalización de la ciencia brasileña^(4)^.

Los programas de posgrado están constituidos por cuerpos docentes, técnico-administrativos y estudiantiles, cuya dinámica involucra múltiples perspectivas y experiencias. La visión y la misión de estos programas exigen de sus actores sociales una reflexión asistencial y gerencial orientada al desarrollo de la ciencia y la tecnología en el área de la salud. Por lo tanto, considerar el desarrollo de estos sujetos se vuelve esencial para comprender los impactos sociales, políticos y culturales generados, lo que contribuye a la mejora y el fortalecimiento de los programas^(4)^.

En Minas Gerais, la Escuela de Enfermería de la Universidad Federal de Minas Gerais (EEUFMG) ha desempeñado, desde su fundación en 1933, un papel central en la profesionalización de la enfermería. Fue responsable de la creación del primer Programa de Posgrado en Enfermería *Stricto Sensu* (PPGE) en el estado, el cual se instituyó en 1993 con la primera promoción de maestría. El doctorado se inició en 2004, ampliando aún más su contribución a la formación de investigadores y al avance científico de la enfermería brasileña^(3)^.

El fortalecimiento de la posgraduación en Brasil se hace evidente a partir de 1981, con la creación del Plan Nacional de Posgrado *Stricto Sensu* por parte de la Coordinación de Perfeccionamiento del Personal de Nivel Superior (CAPES). Actualmente a cargo de la evaluación cuatrienal de los programas, la institución recomienda que, en lo que respecta a la formación, se considere la trayectoria de los egresados, analizando su inserción profesional y los impactos sociales, políticos y económicos derivados de su capacitación^(5,6)^.

Desde 2020, el PPGE/UFMG realiza un seguimiento de sus egresados tras la defensa y durante los cinco años siguientes a la finalización del curso, tal como lo recomienda la CAPES. Se deriva a los egresados al sitio web del programa para que llenen un formulario con información sobre datos de identificación, titulación, formación y desempeño profesional en el período de la Recolección.

A la luz de la sociología de las profesiones, es posible comprender la consolidación de la posgrado en enfermería como parte de un proceso más amplio de profesionalización. Según Freidson, una profesión se estructura a partir de tres pilares fundamentales: la pericia, la autonomía y el credencialismo^(7)^. En este sentido, los PPGEs se convierten en espacios importantes para la constitución de un corpus de conocimiento técnico-científico, contribuyendo a la consolidación de la enfermería como un campo profesional autónomo y reconocido.

La identificación de la trayectoria profesional de los egresados, por lo tanto, es fundamental para evaluar cómo esta formación repercute en su inserción en el mercado laboral, en su prestigio y en su autoridad técnica, reflejando los mecanismos de reproducción y valorización de la profesión en el contexto brasileño contemporáneo.

A pesar de los avances de la posgrado *stricto sensu* en Enfermería en Brasil, aún son escasos los estudios que analizan críticamente los impactos de esta formación en las trayectorias profesionales de los egresados^(8)^. La importancia de las investigaciones en esta dirección se ve reforzada por la Agenda de Prioridades de Investigación del Ministerio de Salud (APPMS), que destaca la necesidad de evaluar el impacto de las ofertas educativas en la cualificación de los profesionales del Sistema Único de Salud (SUS)^(9)^. De manera complementaria, el documento «Prioridades en Investigación para la Enfermería: Propuesta Preliminar» de la Asociación Brasileña de Enfermería (ABEn) señala la relevancia de los estudios sobre la formación de posgrado stricto sensu, su capacidad para desarrollar competencias, calificar el trabajo en salud y contribuir a la gestión del SUS^(10)^.

En este contexto, la comprensión de las trayectorias profesionales de los egresados del PPGE/UFMG en el cuatrienio 2017–2020 se vuelve esencial para sustentar los debates sobre la valorización de la enfermería y el fortalecimiento del sistema público de salud. Ante esto, se plantea la pregunta: ¿cómo se ha perfilado la trayectoria profesional de los egresados del PPGE/UFMG, en el cuatrienio 2017–2020, tras la finalización de sus maestrías y/o doctorados?

El presente estudio tiene como objetivo caracterizar el perfil demográfico, académico y profesional de los egresados de la posgrado *stricto sensu* en Enfermería de la Escuela de Enfermería de la Universidad Federal de Minas Gerais en el cuatrienio de 2017 a 2020, a la luz de la sociología de las profesiones de Eliot Freidson.

Se parte del supuesto de que el título de posgrado stricto sensu en enfermería representa más que una cualificación académica. Actúa como mecanismo de diferenciación en el ámbito profesional, otorgando autoridad técnica y estatus a los egresados. A la luz de la teoría de la sociología de las profesiones, se supone que la posgrado contribuye a la construcción de un saber especializado, legitima posiciones de prestigio en el mercado laboral y refuerza los procesos de profesionalización de la enfermería.

## MÉTODO

### Tipo de Estudio

El presente estudio se inscribe en el campo de la Historia, con una dimensión sociohistórica, orientada al examen de las especificidades de una sociedad, incluyendo los modos y mecanismos de organización social, las relaciones entre grupos, instituciones e individuos, así como los procesos de transformación social^(11,12)^.

La investigación se enmarca en los ámbitos de la historia de la enfermería. El enfoque adoptado es cualitativo y utiliza la Historia Textual, con análisis documental de fuentes escritas.

El estudio se llevó a cabo siguiendo las recomendaciones de la guía *Consolidated criteria for reporting qualitative research* COREQ para investigaciones cualitativas.

### Lugar

El estudio se llevó a cabo en la Escuela de Enfermería de la Universidad Federal de Minas Gerais (EEUFMG), en Belo Horizonte, sede del Programa de Posgrado en Enfermería (PPGE). La delimitación temporal y espacial abarca el cuatrienio de 2017 a 2020, período marcado por un contexto de liberalismo económico, ataques a la democracia y a las instancias de participación social. A este escenario se suma la pandemia de COVID-19, que exigió reestructuraciones en los sectores de la salud y la educación.

### Fuentes y Recolección de Datos

Las fuentes textuales que conformaron este estudio se obtuvieron del Colegio de Posgrado (CPG) de la Escuela de Enfermería de la UFMG, incluyendo la base de datos de egresados del PPGE/UFMG. Se realizaron búsquedas en la Plataforma Lattes, mantenida por el Consejo Nacional de Desarrollo Científico y Tecnológico (CNPq), para recopilar datos sobre la trayectoria profesional de los egresados. La elección de esta plataforma se justifica por ser el principal sistema virtual brasileño de currículos científicos y por tener acceso abierto, lo que permite así la confirmación de la identidad de los egresados.

Se incluyeron como fuentes textuales aquellas que estaban actualizadas y vinculadas al nombre completo del egresado. Se excluyeron los registros incompletos, desactualizados o sin correspondencia verificable con la base de datos del programa.

Los datos fueron recopilados por la investigadora principal entre los meses de octubre de 2024 y abril de 2025.

### Análisis y Tratamiento de los Datos

El tratamiento de las fuentes incluyó la creación y organización de una base de datos en la plataforma Google Drive, descrita y organizada por año (2017 a 2020). Una vez catalogadas, las fuentes se clasificaron a partir de criterios metodológicos establecidos, tales como la pertinencia de las fuentes, la suficiencia, la representatividad, la exhaustividad y la homogeneidad. Las fuentes textuales se analizaron críticamente en cuanto a su veracidad y relevancia, mediante una revisión interna y externa^(12)^. Los hallazgos se analizaron a la luz de la Sociología de las Profesiones de Eliot Freidson.

### Aspectos Éticos

La investigación fue evaluada por el Comité de Ética en Investigación de la UFMG, garantizando el cumplimiento y la adecuación a las normas éticas y legales vigentes. Fue aprobada con el dictamen n.º 7.115.336 en el año 2024.

## RESULTADOS

En el cuatrienio de 2017 a 2020, el Programa de Posgrado en Enfermería de la Universidad Federal de Minas Gerais (PPGE/UFMG) otorgó títulos a 218 estudiantes, de los cuales 130 obtuvieron la maestría y 88 el doctorado.

Del total de egresados, 168 respondieron al cuestionario, lo que representa el 77,06 % del total; de ellos, 87 eran maestros y 81 doctores y de ese total de egresados del cuatrienio, 190 eran mujeres y 28 hombres, lo que evidencia un predominio femenino significativo (87 %). El rango de edad de los egresados del PPGE/UFMG se muestra en el gráfico siguiente (Figura 1), que presenta una mayor incidencia a los 36 años, con 23 egresados, seguido de las edades de 37 (n = 18), 38 (n = 13) y 34 (n = 14) años.

**Figura 1:** Rangos de edades de los egresados del PPGE.

Fuente: Informe de análisis estadístico de los egresados de posgrado (2017–2020), 2025.

La formación inicial de los egresados del PPGE/UFMG está compuesta en su mayoría por enfermeros, con un total de 147 profesionales. El programa también acogió, a lo largo del cuatrienio 2017–2020, a un profesional de Educación Física, uno de Estadística, dos farmacéuticos, tres gestores de salud y ocho nutricionistas, un pedagogo, cuatro psicólogos y un terapeuta ocupacional.

Tras identificar el predominio de enfermeros entre los egresados, se observa, en este grupo, una participación femenina significativa. Los datos del PPGE/UFMG indican que, en el período de 2017 a 2020, 128 mujeres completaron el curso, en contraste con solo 19 hombres.

En cuanto al lugar de formación de los egresados, 62 estudiantes se graduaron en la UFMG, 63 en otras universidades públicas de Brasil y 36 en instituciones de educación superior privadas.

De los 168 graduados que respondieron al cuestionario del CPG, 126 (75 %) poseían un posgrado *lato sensu*, mientras que 42 (25 %) no habían realizado ninguna especialización. En cuanto al lugar de formación del máster, se observó un predominio de la UFMG, con 132 graduados, de los cuales 53 indicaron específicamente la Escuela de Enfermería de la UFMG. También se mencionaron otros lugares, como la Universidad Federal de Paraíba, la Universidad Estatal de Paraíba, la Universidad Federal de Alfenas, la Universidad Federal de Juiz de Fora, la Universidad Federal de Río Grande del Norte, la Universidad Federal de Viçosa, la Universidad Estatal del Suroeste de Bahía (UESB), la Universidad de la Sabana - Colombia, la Universidad Federal de São Paulo (UNIFESP), la Fundación Oswaldo Cruz (Fiocruz) y la Universidad de São Paulo (USP).

Durante la maestría, 38 egresados informaron haber recibido una beca, mientras que 49 cursaron el programa sin ese apoyo. En el doctorado, 49 egresados se beneficiaron de una beca, mientras que 32 realizaron el curso sin recibir esa ayuda. De los 87 estudiantes de maestría egresados del cuatrienio 2017–2020 que respondieron al cuestionario del CPG, 18 continuaron con el doctorado en la EEUFMG, mientras que cinco se dirigieron a otras instituciones como: la Facultad de Medicina de la UFMG (n = 3), las Facultades de Ciencias Económicas de la UFMG (n = 1) y Fiocruz (n = 1).

Antes de ingresar al PPGE/UFMG, la mayoría de los estudiantes ya contaba con diversas experiencias profesionales, lo que refleja múltiples insertaciones en el campo de la enfermería y la salud. Los datos muestran que 59 graduados ya estaban insertados en el mercado laboral antes de la posgrado, mientras que otros 39 iniciaron sus actividades profesionales durante el curso.

Tras la finalización de la maestría o el doctorado, se observa una reducción progresiva en las nuevas incorporaciones profesionales: 28 hasta un año después, 16 entre uno y tres años, y solo nueve después de tres años.

En cuanto a la incorporación al mercado laboral, los egresados muestran que la mayoría trabaja en una sola institución, con 168 encuestados. Un grupo más reducido, compuesto por 33 egresados, trabaja simultáneamente en más de una institución, lo que supone más de una relación laboral. Por el contrario, 17 egresados declararon no estar trabajando en el momento de responder al cuestionario. Solo tres encuestados indicaron que trabajaban de manera autónoma.

En cuanto a los cargos ocupados por los egresados, se observa una diferencia notable entre quienes completaron la maestría y quienes finalizaron el doctorado. De los 88 maestros que respondieron al cuestionario en el período analizado, 43 informaron trabajar en actividades asistenciales, lo que sugiere que muchos egresados mantienen una participación directa en los servicios de salud. Además de este dato, 44 indicaron una relación con la supervisión y la gestión, y tres en puestos de coordinación.

En contraste, en el caso de los doctores, nueve egresados trabajan en la asistencia, 13 desempeñan funciones de supervisión y gestión, 17 ocupan puestos de coordinación, mientras que la docencia se destaca como el principal campo de inserción profesional, siendo mencionada por 64 colaboradores del estudio.

Además de la docencia, también se observa una mayor participación en actividades de investigación y gestión académica entre los egresados del doctorado. De los participantes del máster, 18 declararon estar involucrados en investigaciones, mientras que en el doctorado este número fue significativamente mayor, con 50 encuestados trabajando en el área.

En un universo de 168 encuestados, los datos sobre la ocupación de cargos de coordinación y dirección revelan que, entre los egresados de la maestría, se identificaron cuatro coordinaciones y ninguna dirección, mientras que en el doctorado se registraron 17 coordinaciones y una dirección. De las 21 coordinaciones reportadas por los egresados, 17 están ocupadas por mujeres y cuatro por hombres.

En cuanto al tipo de vínculo laboral de los egresados, los encuestados indicaron que la mayoría (n = 128) trabaja en instituciones públicas, seguidas por el sector privado, con 64 profesionales, mientras que solo cinco egresados trabajan de manera autónoma. En cuanto a sus ingresos, tras el máster, la mayoría de los egresados (n = 22) perciben entre cuatro y cinco salarios mínimos, con ingresos que van desde dos hasta más de nueve salarios. En el doctorado, el perfil cambia: prevalecen los ingresos superiores a nueve salarios mínimos (n = 26), seguidos por los rangos de seis a siete (n = 17) y de ocho a nueve salarios mínimos (n = 16), sin registros por debajo de dos salarios.

En cuanto a la participación de los egresados del PPGE/UFMG en entidades representativas de la enfermería, se observa una baja participación, con solo 23 maestros y nueve doctores involucrados en organizaciones de la profesión.

## DISCUSIÓN

El análisis del perfil de la trayectoria profesional de los egresados del PPGE/UFMG, a la luz de la sociología de las profesiones de Eliot Freidson, permite comprender cómo la formación *stricto sensu* en Enfermería contribuye al proceso de profesionalización y al fortalecimiento del estatus y la autonomía del enfermero en el ámbito profesional.

La edad de los egresados del PPGE/UFMG muestra un perfil similar al encontrado en otras investigaciones nacionales sobre posgrado *stricto sensu*. Estudios como el de Gomes et al.^(13)^, que presenta una investigación con egresados de la Maestría Profesional en Salud de la Familia (PROFSAÚDE), identificaron que el 59,2 % de los encuestados tenía hasta 40 años, mientras que Heinzle et al. ^(14)^ señalaron que el 49,5 % de los egresados de la Maestría en Educación de la Universidad Regional de Blumenau (FURB) tenían entre 31 y 40 años —datos que se asemejan a los hallazgos del PPGE/UFMG.

El carácter multidisciplinario del PPGE/UFMG pone de manifiesto la apertura del programa a la integración de diferentes saberes y prácticas profesionales, consolidándolo como un espacio de construcción colectiva del conocimiento. Esta característica sigue las tendencias de la posgrado en salud global, caracterizada por formaciones interprofesionales y enfoques interdisciplinarios^(15)^. Además, la heterogeneidad del cuerpo estudiantil y las múltiples motivaciones para ingresar, señaladas por estudios recientes, refuerzan la importancia de programas capaces de acoger y potenciar distintas trayectorias académicas y profesionales^(16)^.

Desde la perspectiva de la sociología de las profesiones, las disputas y articulaciones interprofesionales han marcado la historia de la posgrado en Enfermería en Brasil, fortaleciendo su reconocimiento como campo de conocimiento^(3)^. Al integrar a profesionales de diversas áreas, el PPGE/UFMG promueve el intercambio entre profesiones y amplía la producción de conocimiento técnico y científico, elemento central para la consolidación profesional.

Los datos evidencian el predominio femenino en el PPGE/UFMG, lo que refleja el desequilibrio de género históricamente presente en la enfermería. Estudios internacionales señalan una baja participación masculina, como en EE. UU. (9,6 %), en el Mediterráneo Oriental (21 %), en Europa (16 %), en el Sudeste Asiático (21 %) y en el Pacífico Occidental (19 %)^(17,18)^. En Brasil, entre 1950 y 1999, solo el 2,37 % de los graduados de la Escuela de Enfermería de la Universidad de São Paulo (EEUSP) eran hombres^(19)^. Las revisiones indican que el modelo nightingaleano, con fuerte influencia en la enfermería brasileña, reforzó los estereotipos que asocian el cuidado con lo femenino, dificultando la elección y la permanencia de los hombres en la profesión^(20)^.

Se observa un predominio de vínculos con instituciones de enseñanza públicas, con la participación de graduados de diversas universidades públicas del país, lo que refuerza la relevancia nacional del PPGE/UFMG. La escasa presencia de titulados de facultades privadas puede reflejar desigualdades de acceso y diferencias en la trayectoria formativa, que influyen en el ingreso y la permanencia en la posgrado. Según Silva et al.^(16)^, motivaciones como la carrera académica, la cualificación y la profundización de conocimientos se asocian con frecuencia a experiencias de iniciación científica, grupos de investigación y redes académicas vividas en la licenciatura.

Las universidades públicas ofrecen una mayor exposición a la investigación y la extensión, mientras que las universidades privadas tienden a priorizar la formación práctica, lo que puede afectar la preparación para la posgrado^(15)^. Sin embargo, las motivaciones para ingresar no siempre dependen de la institución de origen, sino que abarcan intereses como la especialización técnica, el reconocimiento profesional, la docencia y la identidad académica. Así, a pesar del predominio público en la producción científica, los estudiantes de diferentes formaciones ven en la posgrado stricto sensu una oportunidad de transformación profesional e intelectual^(21)^.

Los datos revelaron que gran parte de los exalumnos del programa ya había cursado especializaciones antes de ingresar a la posgrado stricto sensu, lo que caracteriza una trayectoria formativa progresiva. En otra investigación, realizada por Souza et al.^(22)^, al analizar el perfil de los egresados del doctorado en Enfermería y Salud de la Universidad Estatal del Suroeste de Bahía entre los años 2014 y 2022, se observó que la mayoría de los doctorandos también había realizado cursos *lato sensu* previamente. Así, los datos del PPGE/UFMG confirman que la especialización sigue representando un hito intermedio importante en el recorrido de cualificación de muchos profesionales que buscan avanzar hacia el *stricto sensu*.

A la luz de la sociología de las profesiones de Eliot Freidson, la elevada proporción de egresados con especializaciones pone de manifiesto uno de los pilares centrales de su teoría: el credencialismo, entendido como la adquisición de títulos y certificaciones formales como forma de legitimación del saber profesional^(7)^. La sociología freidsoniana destaca tres pilares fundamentales para el reconocimiento de una ocupación como profesión^(23)^. De este modo, la búsqueda de especializaciones no representa solo un esfuerzo individual por la cualificación, sino un movimiento colectivo de reconocimiento profesional, como lo demuestran los egresados del PPGE, en el que los títulos, es decir, las credenciales, operan como formas de reconocimiento técnico y prestigio profesional, pero también influyen en la ocupación de puestos en el mercado laboral y en la mejora salarial.

En lo que respecta a la dedicación exclusiva, los datos indican que esta no fue la primera opción para la mayoría de los egresados. Este escenario se observa en otros estudios, en los que los participantes de la investigación señalaron la necesidad de mantener vínculos laborales para garantizar el sustento personal y familiar, lo que intensifica la sobrecarga y genera impactos en la productividad académica, el bienestar emocional y las posibilidades de una mayor participación en proyectos de investigación^(24)^.

Así, la situación identificada en el PPGE/UFMG no es un caso aislado, sino que refleja una condición observada en otras instituciones, a la que se enfrentan muchos estudiantes de posgrado en Enfermería.

La inserción profesional de los egresados se produjo en su mayoría antes de ingresar a la posgrado *stricto sensu*. Este dato del PPGE/UFMG concuerda con estudios realizados en Brasil^(25,26,27)^, que señalan a los cursos de posgrado stricto sensu como importantes mecanismos de consolidación y cualificación de las trayectorias profesionales de los estudiantes. De manera similar, en un estudio transversal realizado con estudiantes de doctorado en enfermería en China, se identificó que la mayoría de los estudiantes ya contaba con una inserción profesional previa y buscaba, a través de la formación académica, ampliar sus oportunidades en las áreas de enseñanza e investigación, consolidando así sus proyectos de carrera^(28)^.

Desde la perspectiva de Freidson, estos datos refuerzan la idea de que el PPGE/UFMG actúa como un espacio de profesionalización en el panorama de Minas Gerais y de Brasil más que como una puerta de entrada al ejercicio profesional. Una de las bases del profesionalismo radica en el dominio de un conocimiento esotérico, inaccesible al sentido común, que confiere autoridad técnica y estatus profesional^(22)^. En este sentido, el ingreso a la posgrado *stricto sensu* por parte de actores sociales ya insertados en el mercado demuestra un movimiento de credencialismo, en el que el título académico se busca como instrumento para ampliar la autonomía, el prestigio y la legitimidad en el campo de actuación.

En cuanto a los cargos ocupados por los egresados, se observa una diferencia notable entre quienes completaron la maestría y quienes finalizaron el doctorado. Las respuestas al cuestionario del PPGE/UFMG muestran que la mayoría de los maestros siguen desempeñando funciones asistenciales, mientras que los doctores se concentran más en la educación superior y en la carrera académica. Aunque el título de maestría profundiza la formación teórica y práctica, este avance no siempre se traduce en reconocimiento institucional o en una progresión inmediata en la carrera. Así, la práctica asistencial sigue siendo el eje central de la identidad profesional de los egresados de la maestría, incluso ante la ampliación de oportunidades, como la docencia. Vale la pena reflexionar sobre el hecho de que, probablemente, con la titulación de maestría, este graduado esté más preparado para la atención asistencial. Por su parte, el doctorado, más que la maestría, se ha mostrado como una herramienta potente de inserción en la vida docente, reforzando el papel de la posgrado stricto sensu como una de las principales vías de profesionalización en el campo académico de la Enfermería y de otras áreas de la salud.

En cuanto a los cargos ocupados por los graduados, un hallazgo importante demuestra que la mayoría de los graduados del PPGE/UFMG son mujeres; sin embargo, la obtención de cargos de gestión es proporcionalmente mayor para los hombres en las coordinaciones. Este hecho demuestra una desigualdad de género en el acceso a cargos de liderazgo, incluso en un campo compuesto en su mayoría por mujeres.

La enfermería se considera una profesión femenina, ya que, según Terra et al.^(29)^, esta visión está muy ligada al contexto histórico en el que el cuidado se considera una extensión del trabajo doméstico. Otros estudios señalan que, aunque la enfermería es una profesión con mayor presencia femenina, se caracteriza por relaciones de poder desiguales, en las que los hombres, a pesar de ser una minoría, ocupan con mayor frecuencia puestos de liderazgo y reciben salarios más altos^(29,30,31,32)^.

La disparidad de género es un tema estudiado a nivel mundial y forma parte de los Objetivos de Desarrollo Sostenible (ODS), una iniciativa global lanzada por la Organización de las Naciones Unidas (ONU) como parte de la Agenda 2030 para el Desarrollo Sostenible. La desigualdad de género se aborda en el ODS 05. Por más que la enfermería sea una profesión mayoritariamente femenina, aún presenta desigualdades de género en sus espacios de liderazgo, según lo demuestran los datos de los egresados del PPGE/UFMG. Este dato revela que el discurso de la equidad no siempre se refleja en la práctica. Taminato et al.^(33)^ demuestran que es esencial que el compromiso con los ODS promueva cambios reales en la cultura organizacional de la enfermería, enfrentando las raíces históricas de esta desigualdad.

En cuanto a la remuneración, los datos del PPGE/UFMG indican que aquellos con maestría presentan ingresos medios, mientras que los doctores muestran una inserción más significativa en instituciones de educación superior, investigación y gestión pública, lo que se refleja positivamente en los salarios. Un estudio de la Fundación Oswaldo Cruz (FIOCRUZ) corrobora estos hallazgos, señalando que el título de doctor se asocia con una mayor estabilidad laboral y con la ocupación de puestos estratégicos, especialmente en instituciones públicas, factores que contribuyen a escalas salariales más elevadas, tal como se evidencia en los datos del PPGE^(25)^.

Según la sociología de las profesiones, este fenómeno dice mucho sobre el credencialismo, donde los títulos y certificaciones formales legitiman el conocimiento y diferencian a los individuos en el mercado laboral^(7)^. El título de doctor, como lo demuestran los egresados del PPGE/UFMG, amplía el acceso a puestos más prestigiosos y bien remunerados, como cargos docentes en universidades públicas y privadas y puestos de gestión académica. Así, la ausencia de egresados del doctorado en los niveles de ingresos más bajos corrobora la perspectiva de la sociología de las profesiones, que indica que las credenciales redefinen los puestos profesionales, fortaleciendo el argumento de que el saber científico-académico es un motor impulsor en la búsqueda de estatus, prestigio y mejores condiciones de trabajo.

A la luz de la sociología de las profesiones, las entidades de clase son fundamentales para la profesionalización, ya que legitiman el conocimiento, aseguran la autonomía del ejercicio profesional y protegen los intereses de la categoría, siendo esenciales para consolidar una profesión como de referencia^(7,34)^. Trabajan en pro de la enfermería y de la sociedad y defienden proyectos políticos de formación y cualificación que respondan a los intereses de los profesionales de la enfermería y a las demandas sociales. Representan a los profesionales en los diversos campos de actuación en los que se insertan^(34)^.

La participación de los egresados del PPGE/UFMG en entidades representativas de la enfermería revela un bajo nivel de adhesión. Este bajo número de respuestas relativas a la participación de los egresados del PPGE/UFMG en las entidades civiles de su clase profesional es un dato preocupante, ya que, como lo abordan Carregal et al.^(3)^, la posgrado *stricto sensu* se considera un espacio privilegiado para la producción crítica y el fortalecimiento de la identidad profesional, alineándose con las concepciones freidsonianas de profesionalización.

## CONCLUSIÓN

La posgrado *stricto sensu* reforzó la búsqueda de un conocimiento propio, también llamado isotérico, tal como lo aborda Freidson, que legitima la autoridad técnica de los egresados en sus campos de trabajo. Esta autoridad viene acompañada de la autonomía profesional, sobre todo en la capacidad de liderar proyectos de investigación, conducir procesos formativos e intervenir en los espacios institucionales, tal como lo demostraron varios egresados.

A pesar de los avances formativos y los logros académicos promovidos por el PPGE/UFMG, los graduados también revelan importantes desafíos enfrentados en el proceso de formación, e incluso después de finalizar el curso, que culminan en su trayectoria profesional. Se observaron tanto desigualdades de género como una baja participación en becas de estudio y, aunque las mujeres representan la mayoría entre los participantes de la investigación, ocupan proporcionalmente menos puestos de gestión en comparación con los hombres, lo que corrobora otros estudios.

La experiencia adquirida a través de la titulación de maestría y doctorado no solo fortalece el campo de la Enfermería como ciencia, sino que también amplía sus ámbitos de actuación, incorporando a sus egresados a espacios importantes de toma de decisiones, gestión, investigación y políticas públicas. Sin embargo, existe una baja concientización respecto a la participación asociativa, un punto importante de la profesionalización en la lógica freidsoniana, que merece acciones promotoras por parte de los PPG.

Como todo estudio, esta investigación presenta limitaciones que deben reconocerse, destacando la proporción de egresados que no respondió al cuestionario enviado por el colegio. De este modo, se hace necesario realizar nuevos estudios que acompañen la trayectoria de los egresados a lo largo del tiempo, ampliando la comprensión sobre los impactos de la formación *stricto sensu* en diferentes etapas de la trayectoria profesional. A partir de los hallazgos de esta investigación, se reafirma la relevancia del PPGE/UFMG como espacio formador de actores sociales con conocimientos propios y críticos, comprometidos con la ciencia, la docencia y la sociedad. El perfil de los egresados revela no solo los impactos individuales de la formación académica, sino también los efectos institucionales y colectivos del programa, que se consolida como referencia nacional, como lo evidencia su ascenso a la excelencia.

## Data Availability

Todo el conjunto de datos que apoya los resultados de este estudio fue publicado en el propio artículo.
